# Palliative care in general practice; a questionnaire study on the GPs role and guideline implementation in Norway

**DOI:** 10.1186/s12875-021-01426-8

**Published:** 2021-04-07

**Authors:** Anne Fasting, Irene Hetlevik, Bente Prytz Mjølstad

**Affiliations:** 1 General Practice Research Unit, Department of Public Health and Nursing, NTNU, Norwegian University of Science and Technology, PO Box 8905 MTFS, N-7491 Trondheim, Norway; 2 Unit for Palliative Care and Chemotherapy Treatment, Cancer Department, More Og Romsdal Hospital Trust, Kristiansund Hospital, N-6508 Kristiansund N, Norway; 3 Saksvik legekontor, Saxe Viks veg 4, N-7562 Hundhammeren, Norway

**Keywords:** Palliative care, Primary care, Palliative medicine, General practice, Clinical practice guidelines, Symptom assessment, Advance care planning

## Abstract

**Background:**

Patients in need of palliative care often want to reside at home. Providing palliative care requires resources and a high level of competence in primary care. The Norwegian guideline for palliative care points to the central role of the regular general practitioner (RGP), specifying a high expected level of competence. Guideline implementation is known to be challenging in primary care. This study investigates adherence to the guideline, the RGPs experience with, and view of their role in palliative care.

**Methods:**

A questionnaire was distributed, by post, to all 246 RGPs in a Norwegian county. Themes of the questionnaire focused on experience with palliative and terminal care, the use of recommended work methods from the guideline, communication with partners, self-reported role in palliative care and confidence in providing palliative care. Data were analyzed descriptively, using SPSS.

**Results:**

Each RGP had few patients needing palliative care, and limited experience with terminal care at home. Limited experience challenged RGPs possibilities to maintain knowledge about palliative care. Their clinical approach was not in agreement with the guideline, but most of them saw themselves as central, and were confident in the provision of palliative care. Rural RGPs saw themselves as more central in this work than their urban colleagues.

**Conclusions:**

This study demonstrated low adherence of the RGPs, to the Norwegian guideline for palliative care. Guideline requirements may not correspond with the methods of general practice, making them difficult to adopt. The RGPs seemed to have too few clinical cases over time to maintain skills at a complex and specialized level. Yet, there seems to be a great potential for the RGP, with the inherent specialist skills of the general practitioner, to be a key worker in the palliative care trajectory.

**Supplementary Information:**

The online version contains supplementary material available at 10.1186/s12875-021-01426-8.

## Background

### Palliative care and general practice

In recent years, there has been an increasing need for palliative care, both due to demographic changes increasing the amount of elderly and multimorbid patients, and to the success of modern cancer treatment increasing longevity [1, 2]. Most patients with palliative needs want to be cared for, and possibly die, in their own homes [3–5]. In Norway, less than 15% die in their own home [2]. The term “palliative care”, is defined by the European Association for Palliative Care (EAPC), as the total care of patients with incurable, life threatening disease:

*“Palliative care is the active, total care of patients whose disease is not responsive to curative treatment. Palliative care takes a holistic approach, addressing physical, psychosocial and spiritual care, including the treatment of pain and other symptoms. Palliative care is interdisciplinary in its approach and encompasses the care of the patient and their family and should be available in any location including hospital, hospice and community.*

*Palliative care affirms life and regards dying as a normal process; it neither hastens nor postpones death and sets out to preserve the best possible quality of life until death.”* [6]

This means that not only cancer patients, but all groups of patients with life limiting disease, can benefit from the interdisciplinary approach of palliative care, even early on in the trajectory of the disease [1, 7].

The characteristics and core values of palliative care have many parallels to the person centered and holistic approach of general practice as described in the European definition of family medicine [8]. The World Health Organization (WHO) explicitly lists palliative care as one of the general practitioner’s (GP’s) tasks [9].

Thus, the GP should be well situated to contribute in the palliative care trajectory. Through the regular general practitioner (RGP) scheme in Norway [10], all residents are entitled to a RGP that is responsible for the coordination of medical care. At the time of the study, more than 99% of the Norwegian population was listed with an RGP [11].

### The Norwegian guideline for palliative care

Alongside the increase in patients in need of palliative care, there has also been a shift of focus; from care given in institutions, towards care given at home for these patients [4, 12, 13]. This is demanding for the primary care services. The Norwegian guideline for palliative care comprises recommendations for treatment of specific symptoms, standards for organization of the service and competence requirements. Although the guideline is said to be relevant for all patients with life limiting disease, its origin and organization is within the Norwegian national program for cancer care [1], and the patient population within the palliative care units is described as consisting of 95% cancer patients [14]. The guideline attachment addressing organization and competence requirements was authored by a majority of hospital specialists, but also a nurse, and one specialist of general practice, working in a palliative care unit [14]. The guideline assigns the RGP a key role [1]; The RGP is supposed to coordinate all patient care, make house calls, and make an individual plan for how the patient can access medical help outside office hours, and when the RGP is absent. The guideline specifies a level of competence for all physicians who are involved in palliative care, including the RGPs. This level includes advanced procedural skills such as the use of specific symptom assessment-tools, e.g. the Edmonton Symptom Assessment System (ESAS-r) and mastering the principles of using a syringe driver for subcutaneous administration of medications. The RGP is also expected to work proactively towards involving other professionals when needed, such as specialized community nurses or hospital specialists. Advance care plans (ACP) and updated medical information should be available to all personnel involved with the patient. The use of joint meetings between hospital specialists and the RGP, ahead of discharge from hospital, is also strongly recommended [1].

Previous reports support that the RGP should adopt a central role in this work; the need for a coordinator has been demonstrated, and there also seems to be an association between the RGP making house calls and the patient being able to die at home [15–20]. However, previous findings indicates that there seems to be a discrepancy between the RGPs’ actual clinical practice, and what the health authorities recommend in the guideline [1, 21, 22].

### The use of guidelines in clinical practice

Clinical guidelines are important means for directing health care resources towards evidence-based practices [23]. Previous studies suggest that GPs have difficulties in adhering to guidelines in different fields [24–29]. According to the European Science Foundation, the process is working well from the initial idea, through research, meta-analysis, and Cochrane Review. The problem arises, however, because: “*The process from meta-analysis through guidelines to clinical practice is a source of considerable variation throughout Europe and therefore suffers from non-transparency and fragmentation*”[30].

As far as we know there has not been conducted any Norwegian study that has examined the extent to which the RGPs’ practice comply with the guideline. The aim of this study is therefor to investigate the RGPs’ adherence to the Norwegian guideline for palliative care. The questionnaire (Supplementary file 1) encompasses questions related to knowledge about, and attitude towards the guideline as well as self-reported experience and clinical practice in palliative care, all elements useful in the study of guideline implementation [30]. By creating a questionnaire exploring the RGPs’ experiences with palliative care, we can also get information about the clinical reality in which the guideline is meant to be implemented. The study will contribute to information that might be important regarding both the development and implementation of guidelines in primary care, and to inform future organization of the palliative care service.

## Methods

As no suitable, validated questionnaire could address our research question, we created a questionnaire based on elements drawn from the national guideline for palliative care (Supplementary file 1). Relevant topics from the guideline regarding the RGPs competence and role were chosen by the authors and validated by peers and one hospital specialist in palliative medicine. The questionnaire was sent by post to all 246 RGPs in the Norwegian county of Møre og Romsdal in 2014. The population of the county was approximately 250 000. The chosen county includes both rural areas with scarce populations, and urban districts with larger towns. The county has four local hospitals of different sizes. A reminder was sent to all RGPs two months after the original deadline. All answers were anonymous.

Most of the questions were related to themes in the guideline, particularly concerning the organization of the palliative care service, specific competence requirements for RGPs, and procedures of cooperation. The RGPs also answered questions related to their personal experience with palliative care and their understanding of own role, as well as their participation in terminal care at home. The questions were partly "yes / no", and partly 5-point Likert-type questions, ranging from "agree” to “disagree", as well as questions with fewer options or numeric information. Themes of focus were: “experience with palliative and terminal care”, “use of guideline recommended procedures”, “communication with partners”, “RGP role” e.g. sense of being central participant in palliative care, and “confidence” in palliative care. The questionnaire items included both positive and negative statements for balance (Supplementary file 1). Nine of the respondents had chosen to answer the questionnaire as a group, using one form. Although their responses are included in the descriptive part, they were excluded when describing differences between subgroups. The form also provided space for freely written comments. Frequency analysis was performed by using the software SPSS statistics 25. All written comments in free text were analyzed for content.

## Results

**Fig. 1:**
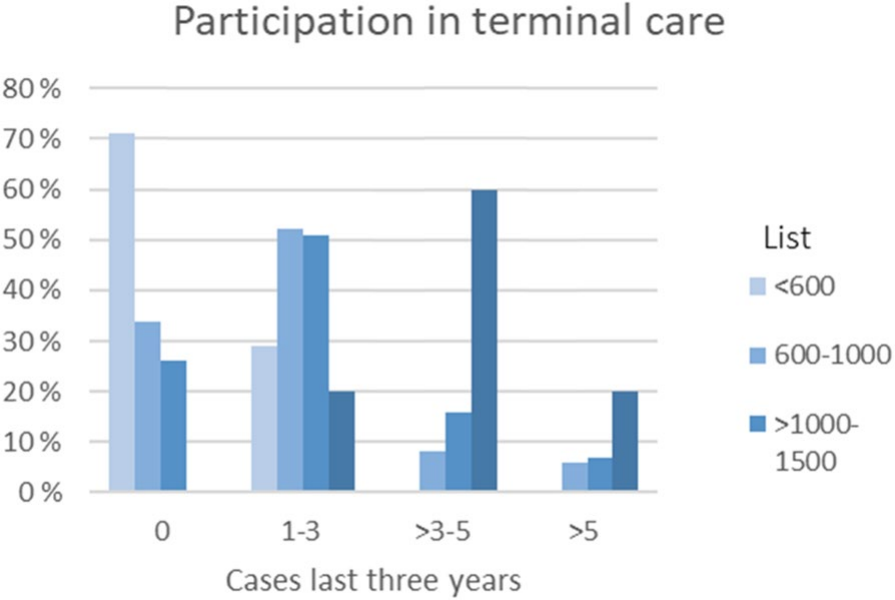
participation in terminal care increased with increasing patient list of the RGP

The response rate was 57%, as 142 out of 246 RGPs responded (Table 1). All participants worked in positions as RGPs, 8% being temporarily employed (locums). RGPs affiliated with all hospitals in the county, participated in the study. For half of the respondents (51%), the distance to hospital was less than half an hour. RGPs reporting to be affiliated with a hospital outside the county, all had more than 30 min or more travel distance to hospital. Most RGPs (91%) had between 600 and 1500 patients listed. Among the 41% of the RGPs also working as nursing home physicians, there was no significant geographic variation. Participant information is listed in Table 1, main results in Tables 2, 3 and 4, and Fig. 1.

**Table 1:** RGP characteristics

**Total number responders n (%)**	142 (100)
Regular general practitioner	130 (92)
Locum	12 (8)
**Local Hospital**	
Ålesund	46 (32)
Molde	39 (28)
Kristiansund	23 (16)
Volda	28 (20)
Other	6 (4)
**Distance from local hospital**	
< 30 min	73 (51)
30 min – 1hour	38 (27)
> 1 h	31 (22)
**Size of patient list**	
< 600	9 (6)
600 – 1000	66 (47)
> 1000 – 1500	62 (44)
> 1500	5 (3)
**Combined work as nursing home doctor**	54 (41)
1 – 4 h/week	19 (35)
5 – 7,5 h/week	24 (44)
> 7,5 h/week	11 (20)

**Table 2:** Guideline recommended procedures

	N (%)		
**Use of guideline recommendations**	**Agree**^**a**^	**Neutral**	**Disagree**^**b**^
I use forms for symptom assessment regularly	28 (20)	22 (16)	90 (64)
The use of such forms is unknown to me	40 (29)	19 (14)	80 (57)
The district nurses use such forms	41 (30)	25 (18)	72 (52)
I rely on forms for clinical decisions to a high degree	20 (15)	27 (20)	89 (65)
Palliative patients always have an ACP	32 (23)	37 (28)	67 (49)
Updated information always in patient’s home	38 (28)	30 (22)	67 (50)
Medical information rarely available in patient’s home	77 (57)	23 (17)	36 (26)

Likert-type questions for the use of guideline recommended procedures, N of respondents (valid %),

^a^ Agree fully or partially, ^b^ Disagree fully or partially

**Table 3:** self-reported role of the RGPs

	N (%)		
**RGP role as central in palliative care**	**Agree**^**a**^	**Neutral**	**Disagree**^**b**^
Central worker in palliative care	72(52)	40(29)	27(19)
Palliative patients consult regularly for pain relief	77(55)	33(24)	30(21)
Patients are mostly handled by specialists	52(37)	30(21)	58(42)
Patients do not need me, due to specialist involvement	22(16)	31(22)	86(62)
Available outside office hours when patient is palliative	65(47)	27(20)	45(33)
Specialists dictates treatment, RGP writes prescriptions	53(38)	29(21)	57(41)

5-point Likert-type questions for the RGP role as central in palliative care, N of respondents (valid %)

^a^ Agree fully or partially, ^b^ Disagree fully or partially

**Table 4:** RGP confidence in palliative care

	N (%)		
**RGP confidence in palliative care provision**	**Agree**^**a**^	**Neutral**	**Disagree**^**b**^
I have sufficient knowledge of palliative care	58(41)	47(33)	37(26)
I feel secure in providing palliative treatment	79(56)	31(22)	31(22)
It is difficult to provide palliative care in general practice	28(20)	36(26)	76(54)
I need to improve my knowledge of palliative care	113(80)	17(12)	11 (8)
I feel insecure in the provision of palliative care	30(21)	36(26)	75(53)

Likert-type questions for the RGP confidence in palliative care provision, N of respondents (valid %),

^a^ Agree fully or partially, ^b^ Disagree fully or partially

### RGP experience with palliative and terminal care

Close to one third of the RGPs (32%) reported not to have any patients with need for palliative care at the time, 39% had 1–2 such patients, and only 6% estimated to currently have more than 5 palliative patients. Answers also indicated that they did not see all these patients regularly.

Only a minority (18%) of the RGPs agreed with the statement: “I see enough patients with palliative needs to maintain my competence in palliative care”, whereas the majority (57%) disagreed.

One third (35%) of the RGPs had no experience with terminal care at home within the last 3 years, about half (47%) had been involved in only 1–3 such patients, and some (12%) had experienced 4–5 cases. Very few (6%) reported involvement in more than five patients over the last three years. Frequency of RGP participation in terminal care increased with increasing size of patient list, with 60% of RGPs with patient lists > 1500 patients reporting participation in 3–5 cases, and none of them reporting never to have participated in the last three years (Fig. 1).

Twenty-one of the RGPs (6%) had experienced that patients had not been able to die at home despite wishing to do so. Consideration for relatives (48%), inadequate symptom control (41%), and acute complications that could not be handled at home (48%), were most frequently acknowledged as contributing reasons for not achieving the patients’ goal of dying at home.

The RGPs made several written comments regarding the amount of experience they had in palliative care;

- *“I have few patients needing palliative care, and this makes it difficult to get enough practice/experience”*
- *“Due to having so few patients, for instance only one on a syringe driver, I can’t be updated on this. The palliative teams are important! There are many areas to keep updated on”*
- *“Most of these patients end up in institutions and I don’t see them”*
- *“Too many questions [in the questionnaire] about palliative care, considering that we have so few palliative patients! My experience with palliative care comes mainly from the nursing home”*

### The use of guideline recommended procedures and cooperation with partners

Details of the five-point questions for the use of guideline recommended procedures, reflecting guideline adherence, are given in Table 2. We found no difference in answers related to size of patient list, distance from hospital or RGP also working in nursing home.

Only one fifth of the RGPs use other assessment tools than the Visual Analog Scale (VAS), like the ESAS-r regularly, and approximately half of the responders reported that the district nurses did not use symptom assessment forms to report symptoms either. The RGPs largely agreed to the usefulness of both symptom assessment forms and the sharing of updated information and ACPs. Still, only a few based their treatment on such forms, and half reported that their patients did not always have an ACP available to all levels in the healthcare service. Most agreed that they based palliative treatment on dialogue with the patient and previous knowledge of the situation. Nearly 60% of the RGPs reported that they had too few clinical cases for the symptom assessment forms to be useful to them.

Close to 70% of the respondents had never participated in a joint discharge meeting regarding patients needing palliative care, and only one respondent reported participating in such meetings regularly.

Most of the respondents (60%) agreed that communication between the healthcare service levels worked well, and that specialists and palliative teams were easily accessible for advising decisions (66%). Around 65% of the RGPs agreed that hospital specialists had a good understanding of the working methods and available resources in primary care. Most of the respondents (85%) agreed that the community care services followed up these patients adequately, and 72% reported that their palliative patients mainly achieved good symptom relief.

There were some written comments on the item of symptom assessment tools, several conveying a reluctance towards assessment forms for palliative patients, both regarding ethical issues, due to time constraints, or arguing for a different approach all together:

- *“There is a demand for effectiveness, and no time for unnecessary procedures”*
- *“I think using [assessment] forms take a lot of time”*
- *“I’m sure these forms would be useful, had I known about them”*
- *“Palliative patients have limited resources, and it is unethical to bother them with such procedures”*
- *“In my experience, palliative care requires creativity, and medicine by “recipe” works particularly badly for this patient group”*
- *“I make the plans for the patient and assess the symptoms as we proceed in our conversation”*

### The RGP role in palliative care.

Details of the items about the self-reported role of the RGPs are given in Table 3

About half of the responders reported that they were central workers in palliative care for their patients and about the same proportion reported to make themselves available outside their work hours when a patient is in the palliative setting. RGPs with more than half an hour distance from hospital more often agreed that they made themselves thus available (68%) than RGPs with less than 30 min distance (30%). They also to a larger extent reported to be central workers in palliative care, and to a lesser extent reported that patients were mostly handled by specialists, and that they did not need the RGP.

The participants wrote several comments on this topic, highlighting different, opposing views; on the one hand, several wrote that they do prioritize these patients and make house calls outside work hours, yet others argued strongly against making themselves available out of hours:

- “*I prioritize these patients and make house calls after my regular office hours”*
- *“It is ridiculous to expect the GP to be available 24 h a day”*

### RGPs confidence in providing palliative care

Details for the items regarding the RGPs’ confidence in the provision of palliative care is given in Table 4.

Most RGPs reported to be secure in the provision of palliative care and did not find it difficult to provide such care in general practice.

## Discussion

### Main findings

We found that each RGP had few patients needing palliative care and that they also had little experience with terminal care in the patient’s home. Limited experience challenged the RGPs’ possibilities to maintain advanced knowledge and skills in palliative care. Their clinical approach towards palliative care did not comply with the guideline; although the RGPs largely agreed to the usefulness of the recommendations, they did not use, and seemed unfamiliar with important work methods described in the guideline. Yet, most of the RPGs reported to see their role as central and seemed confident in the provision of palliative care. RGPs sense of centrality in the palliative trajectory was larger for those RGPs situated more than 30 min from hospital.

### Strengths and limitations

The questionnaire (Supplementary file 1) opens for the possibility of biased self-reporting, leading participants to give exaggerated accounts of socially desired behavior [31]. This may also be a strength as there is no reason to suspect that the participants would report knowledge and skills they do not possess. The total anonymity of the survey could mitigate this bias, by allowing the respondents to express themselves more freely. Due to the importance of knowing distance from and affiliation to local hospital, information on age and gender of participants was not included in the survey, as these data could lead to identification of certain RGPs. The material gives no information of how these factors influences the answering, and challenges external validity. A response rate at 57% must be regarded as a strength as all RPGs in the county were invited, and GPs are known to typically have low response rates [32]. The non-responders (43%) may, however, present problems of participation bias, with the risk of failing to capture the full range of views. One could suspect non-responders possibly to find the topic of palliative care less relevant, and to be less active and interested in the subject of study than those who did answer the survey, thus causing over-estimation of experience and knowledge among the RGPs [33]. It has also been shown that GPs are less likely to respond to a survey the more time has passed since qualifying as doctors [33]. This may have caused more experienced RGPs not to answer, thus causing an under-estimation of the amount of experience and skills of the RGPs in our material. A previous study has shown such a positive relationship between age of the GP and both confidence about being a key worker, and likelihood of providing end of life care [34]. The total anonymity of respondents may have led to inappropriate mailing to RGPs that had already answered, causing some to answer the questionnaire twice. However, it is unlikely that many have taken the time to do this, especially as the reminder was sent shortly after the original deadline. Written comments were optional in the questionnaire and only a few respondents used this opportunity (Supplementary file 1). This may have resulted in only respondents with strong opinions commenting, and thus the results may not be representative for the total group. We still chose to include some comments in our results, thinking they convey attitudes and thoughts, apt to help in the interpretation of our findings. Although some missing data, this only caused minor alterations to our frequencies percentages results, and these are therefore given as valid percentages of those who answered. Due to sampling being restricted to one county, caution must be taken when generalizing from our findings. However, we do believe that the geographic spread of participants within the county is indicative of its representability. The area contains four hospitals of differing size, and is typical for many Norwegian counties, although lacking a larger university hospital. The data was collected in 2014. As there has not been structural changes to the palliative care services, competence requirements or general practice in Norway [1, 10, 35], we have no reason to think that our main findings are no longer valid.

### Findings in the light of current knowledge

Achieving death at home for those who wish is in many respects an ideal in palliative care [36] and GP participation in the trajectory is one of many factors identified as facilitators for achievement [15, 16]. The competence requirements and role assigned to the RGPs in the guideline is a means to govern RGP participation in the wanted direction [30, 35]. We found that one third of the RGPs had not participated in terminal care at home the last three years, and that most who had participated had only experienced a few cases. The proportion of patients dying at home yearly in Norway is about 15% or less [37], and a recent study showed that the potentially planned home deaths in Norway were 6.3% of all deaths [38]. This means that an individual RGP will potentially experience a home death in their population, on average, about every two to three years, perhaps even less, as we found that near 40% of RGPs perceive that these patients are mainly handled by hospital specialists. Furthermore, we found that seeing few patients with palliative care needs, challenges the RGPs ability to maintain their competence in the field at the level required by the guideline. The finding is consistent with previous studies of procedural skills practice and training [39–41] and in agreement with a previous Norwegian study by Austad et al. [29], who found that GPs find it difficult to keep updated on guidelines for specific diseases that they do not see regularly.

The low guideline adherence among RGPs is also in agreement with previous studies of guidelines. There is a debate as to whether this may be due to lack of willingness of the GP [27], or to the guideline content [25, 28]. Comprehensive guidelines, also makes it difficult for the GPs to adhere [29], and the GPs' situation of having multiple guidelines to follow simultaneously has been identified as one factor that may impede guideline adherence [29]. The guideline for palliative care, however, differs from previously studied guidelines as it can be viewed as not diagnose-specific, thus representing a common pathway for multiple diseases at the end of life [1]. Hence, there should be no mismatch between guideline and patients’ needs due to multitude of guidelines for single diseases, as previously described [29]. Still, the RGPs seem unfamiliar with the contents of this guideline. Paradoxically, although recognizing the utility of forms like the ESAS-r for symptom assessment, the RGPs seemed to be reluctant to use them. They also seemed to recognize the utility of ACPs and available, updated medical information, but did not use them either. These paradoxes seem parallel to previous findings [27]; the GPs report to acknowledge the value of guidelines, yet seeming unable to use them, and the relevance of guideline content itself may be questioned [27]. Our data implies that the RGPs are not able to meet the competence requirement and maintain the skills they are expected to in the guideline, and it needs to be established how this affects the cooperation and division of labor within the health care services.

Our findings also indicate that the RGPs to a certain degree actively choose a different approach for various reasons. They seem to perceive that they have too few cases for the symptom assessment forms to be useful to them. At the same time they confirm that they commonly approach their patients through conversation and make use of their previous knowledge of the individual patient, consistent with the widely used patient-centered clinical method of general practice described by Levenstein et al. [42]. A Norwegian study has shown that RGPs, and especially experienced RGPs, also rely strongly on person-related knowledge about their patients and that too much standardization in patient care plans can hinder genuinely tailored, individual treatment [43]. This may indicate that the working methods described in the guideline, based on the specialist health services' way of doing it, do not harmonize with the more flexible person-centered approach and working methods in general practice, demonstrated in a previous study [44].

The GP as entry-point and coordinator of primary care is a trait shared by many European countries [45] and palliative care is one of the core values of general practice according to the WHO [9]. Our findings may therefore be relevant to European and other countries with a similar health care structure. Implementation of generated medical knowledge by means of clinical guidelines is a widespread strategy in the world today and understanding of the barriers for implementation is important [30]. Our findings challenge guideline content with respect to the complexity of the knowledge the RGP is expected to maintain when patient encounters are infrequent, and whether the recommendations in the guideline fits the working methods of general practice. The guideline [1] could represent a common pathway for several diagnoses, but as it describes the patient population as consisting mainly of cancer patients, together with its origin and formal organization within the cancer care program, it may not seem relevant for general practice [1]. Whereas cancer seem to be the most frequent patient group from the point of view of the specialist in palliative care units, frailty, organ failure and dementia dominate causes of death in primary care, confronting the GP with a large variety of trajectories [46], that perhaps are difficult to standardize, as pointed out in a recent editorial by Mitchell and Murray [47]. This also raises the issue of guideline applicability as a barrier for adherence [48].

We found that only one respondent reported to participate regularly in joint meetings with the hospital specialists upon discharge from hospital, and that nearly 40% of the RGPs perceived that hospital specialists mainly handled their palliative patients. Discharge planning is an important task for the hospital based palliative teams [49]. A customized approach is expected to be beneficial and should incorporate a clear “care transition” [50]. The finding may imply that the specialist level do not act according to the guideline recommendations either [1]. This is in agreement with a previous implementation study, who demonstrated low guideline adherence among hospital specialists when they were supposed to hand over tasks to GPs [51].

In 2017, an evaluation of the palliative care services in Norway was performed [52], the report describing the RGP as “on the sideline” of palliative care. Our finding that only one fifth of the RGPs do not see themselves as central in this work, and that most seem confident in the provision of palliative care, contrasts somewhat with this report. The finding that the RGPs to a high degree make themselves available out of normal work hours also challenges this report. To our knowledge, no other Norwegian health worker has been shown to make themselves available, in their spare time, and to such an extent, and this comes in addition to having high reported work hours in the first place [53]. These findings are consistent with previous findings of GPs’ commitment towards cancer patients, and providing palliative care [44, 54].

The impact of distance from hospital on the RGPs perceived role in palliative care is an interesting finding. A previous Norwegian study found that that some rural and small-town GPs contributed considerably to cancer care in their patients’ local communities [55]. In a recent Danish study, they found that rural GPs were more secure in the administration of subcutaneous medication than their urban colleagues [34], and in a Dutch study, rurally based GPs were more confident in administering emergency care than urban or metropolitan GPs [56]. In the latter study, this was perceived as linked to proximity to the hospital emergency services, leading to the urban GPs being surpassed. In the case of palliative care, the hospital based palliative teams in Norway are meant to be ambulatory, acting as consultants supporting primary care [1]. The teams should ensure equality of services regardless of geography, which is a widely recognized principal in health care organization [57]. Although cultural differences between rural and urban RGPs may contribute [56], our finding may also represent a distance decay effect [58], pointing towards the possibility of unwarranted variation in the specialist service provision.

## Conclusions

RGP participation in the palliative care trajectory is important to achieve the goals set by the Norwegian health authorities. Still, the RGPs display low adherence to the national guideline and have not adopted the working methods recommended in palliative care. Reluctance towards symptom assessment forms and ACPs despite judging them useful, may indicate something more than unawareness of guideline content; The guideline recommendations, inherently based on the specialists’ view of best practice, may not correspond with the existing working methods of general practice, making them difficult to adopt in the clinical reality of the RGP. Clinical experience is important, and the mismatch between guideline and practice in our study may thus be at least partially explained by the fact that the RGPs have too few clinical cases over time to maintain skills at a complex and specialized level. The competence requirement posed on the RGPs in this specific guideline, may thus be difficult to implement in general practice. It is also a paradox that as much as half of the RGPs see themselves as central, at the same time as public evaluations see them as missing in the trajectories. Our findings indicate a great potential for the RGP, contributing with the inherent skills and working methods of the specialty of general practice, to be a central, key worker in palliative care.

## Supplementary Information

**Additional file 1.** Questionnaire translated to English. The questionnaire consists of the first part of the original questionnaire and contains all questions relevant for this article. The original questionnaire also comprised a separate section about education, not relevant for this article, and not included in the supplementary file.

## Data Availability

Data could be available from the corresponding author on reasonable request.

## Abbreviations

ACP: Advance care plan
ESASr: Edmonton system assessment system, revised
EAPC: European Association for Palliative Care
GP: General practitioner
RGP: Regular general practitioner
VAS: Visual analog scale
WHO: World Health Organization
