# Supplementary material for: Palliative care in general practice; a questionnaire study on the GPs role and guideline implementation in Norway
Source: BMC Fam Pract. 2021 Apr 7;22:64. doi: 10.1186/s12875-021-01426-8 (PMC8028821; doi:10.1186/s12875-021-01426-8)
Supplement: Supplementary file 1 — Additional file 1. Questionnaire translated to English. The questionnaire consists of the first part of the original questionnaire and contains all questions relevant for this article. The original questionnaire also comprised a separate section about education, not relevant for this article, and not included in the supplementary file. [file 12875_2021_1426_MOESM1_ESM.docx]

Regular general practitioners (RGP) and GP locums should answer the questionnaire. Interns should not answer

**General information:**

1. **Are you RGP or locum?**


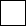


**
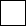
**

RGP

Locum

How long have you been a locum in this practice?


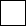


**
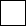
**

**
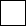
**

0-6 months

> 6 months - 1 year

> 1 year

**2: Which is primarily your local hospital**


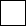


**
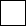
**


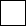

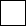


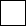


Kristiansund hospital

Molde hospital

Ålesund Hospital

Volda Hospital

None of the above

Remarks:__________________________________________________________________

**3: What size is your list of patients?**


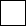


**
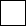
**


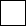


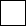


Up to 600 patients

600 - 1000 patients

>1000-1500 patients

> 1500 patients

**4a: Do you also work as a nursing home physician?**


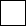


**
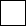
**

No Skip to qu. 5

Yes -----> **4b: how many hours a week?**

1-4h/week


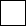


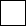


**
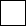
**

5-7,5h/week

> 7,5h/week

**5:** **How many patients needing palliative care do you think there is in your patient population now?**

I think i have
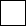
 no. of patients with a need for palliative care now

**6: How many of these patients do you see regularly at consultation or home visits?**

I have
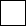
 patients needing palliative care that I see regularly

**7: Which of the following hospitals do you work with when it comes to palliative care? Om check**


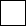


**
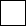
**


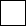


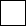


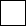


Kristiansund Hospital

Molde Hospital

Ålesund Hospital

Volda Hospital

None of the above

Remarks:___________________________________________________________________

**8:** **How long travel distance is it to hospital from your community?**


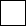


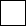


**
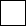
**

< 30 min

30 min -1 hour

>1 hour

**9: Is there a district cancer nurse in your community?**


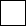


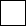


**
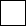
**

Yes

No

I don’t know

**10: Are you familiar with «standard for palliasjon» from 2004?**

Yes, extensive knowledge


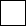


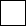


**
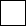
**

**
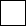
**

Yes, roughly

Yes, I know of its existence

No

**11: Have you taken courses in palliative care the last 5 years?**


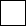


**
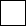
**

Yes

No

**12: Are you planning to take a course in palliative care within the next year?**


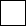


**
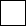
**

Yes

No

**Remarks:____________________________________________________________________________**

**______________________________________________________________________________________**

**______________________________________________________________________________________**

**______________________________________________________________________________________**

**______________________________________________________________________________________**

**In this part of the questionnaire, we ask you to answer in which degree the statements apply to you. One checked box for each line.**

**13: Symptom assessment**

*How are symptoms and complaints assessed in patients with needs for palliative care in your practice? Several standardized forms are in use in the hospitals and palliative teams, that assess more dimensions than the frequently used Visual analogue scale (VAS). Examples is the Edmonton Symptom Assessment System (ESAS)- and trajectory forms. Little is known about the use of such forms in primary care.*

Agree Partly agree neutral Disagree partly Disagree


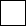

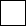

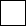

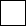

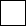


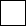

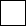

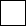

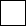

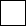


I use forms for symptom assessments

(except VAS) systematically and regularly

The use of such forms is unknown to me


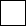

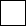

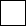

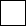

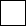


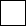

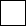

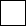

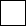

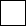


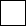

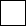

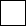

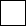

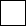


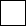

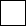

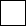

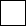

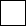


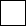

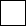

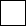

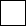

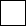


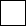

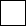

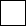

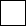

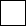


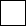

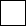

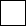

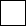

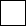


District nurses use such forms

and report the results to me

Such forms make it easier to provide

good symptom relief

Such forms have little value for me as a GP

I use those forms so seldom, or have so

few clinical cases, that the value is low

I rely on forms for clinical decisions to a

high degree

I base decisions for treatment mainly

on previous knowledge and talking to -

the patients

Personal knowledge and the VAS are

sufficient to assess symptoms and

complaints for my use

Remarks: ___________________________________________________________________________

______________________________________________________________________________________

______________________________________________________________________________________

_____________________________________________________________________________________

**14: Symptom relief**

*Relieving symptoms in the palliative stage of disease, is very important. This concerns the daily life, in which pain, gastrointestinal problems, breathing problems etc., can be problems, as well as in emergencies when there are acute complications to the condition.*

Agree Partly agree neutral Disagree partly Disagree


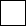

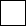

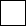

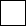

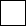


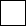

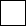

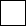

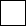

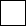


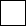

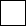

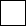

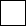

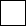


In my experience, patients needing palliative

care mainly receive good symptom relief

Emergency services works well when it comes

to palliative care in my community

I find it difficult to provide palliative care

in general practice

In my experience, patients do not receive

adequate palliative care in situations that

arise outside my working hours

The district nurses adequately follow up

on my patients needing palliative care

**15: The role of the GP in palliative care**

Agree Partly agree neutral Disagree partly Disagree

I have sufficient knowledge of palliative care

I see enough palliative patients to maintain

my competence in palliative care

I need to improve my knowledge of palliative

care

My patients in the palliative stage frequently

consult me when it comes to pain and

symptom relief

Palliative care is mostly handled by

specialists, and I most often isn’t involved

In my experience, the specialists control the

treatment and I just write prescriptions they

have ordered

I have enough time within my working hours

to make house calls to my patients needing

palliative care

I think palliative patients do not need me

because specialists are highly involved,

and this is enough for the patient.

I am a central worker in palliative care

of my patients

I feel secure in providing palliative treatment

I feel insecure in the provision of palliative

care

My patients needing palliative care want

me to be available outside working hours

District nurses expect me to be available

outside working hours

I make myself available outside working

hours for palliative patients and their

relatives

I want a more central role in palliative care

for my patients

**16: Documentation and communication between professionals**

**16a: To what degree do the statements apply to you? Check one box for each question**

Agree Partly agree neutral Disagree partly Disagree

Palliative patients always have an

advance care plan available for all levels

of care

My palliative patients always have a home

journal, with updated medical information

available in their home

I have no experience with advance care

plans or the keeping of home journal

Keeping a home journal with the patient is

an important tool in the case of emergency

Home journals are rarely available/used

Home journals should be used more often

Communication between care levels work

well

Discharge papers and other communication

from hospital or palliative team are often late

It is easy to get hold of the hospital

specialists or the palliative team for advice

so that problems can be solved without

hospital admission

I often consult a specialist or the palliative

team

Consulting with specialists or the palliative

team prevent unnecessary admissions

Hospital specialists and the palliative team

have a good understanding of the work form

and available resources in primary care

The hospitals and/or the palliative team

have unrealistic expectations of the GP

and district nurses’ resources

**16b: Have you participated in joint meetings with the hospital specialists ahead of discharge for patients needing palliative care at home?**

Never

Rarely

From time to time

Regularly

**17: Terminal care**

*Several studies show that many that die want to do so in their own home. Still, a great majority die in institutions (hospitals, nursing homes, hospices) What GPs experience as promoting or inhibiting home death is of interest.*

**17a: How often have you been the physician in a planned death at home among your patient population in the last 3 years?**

Never

1-3 cases

>3-5 cases

>5 cases

**17b: Have you, in the last 3 years, experienced that home death has not been achieved despite the patient wishes?**

No

Yes

If «yes», to what degree do you think the following factors contributed to this?

High degree Some Low degree

Patient changed opinion

considerations for the relatives

Single patient, no relatives in the home

Inadequate symptom control in the home

Acute complications that could not be handled

Patient comorbidity

Insufficient resources of district nurses’ service

Insufficient competence in the distr. nurses’ service

Admission taking place outside GPs working hours

Emergency service physician had inadequate insight

Emergency service physician was inexperienced

with palliative care

Inappropriate communication between care levels

Lack of support/advisory service to GP/nursing service

Insufficiency or lack of ACP

No home journal with updated medical information

I didn’t have sufficient resources to follow up to the

extent needed.
